# Research on predicting the state of health of lithium-ion batteries via back propagation network based on multi-feature combination of electrochemical impedance spectroscopy

**DOI:** 10.1371/journal.pone.0354706

**Published:** 2026-08-06

**Authors:** Fei Chen, Shulei Sun, Xiuxian Jia, Zhong Ren, Xiaorong Huang, Xianan Shu, Jun Li

**Affiliations:** 1 School of Automobile and Transportation, Xihua University, Chengdu, China; 2 Sichuan Engineering Research Center of New Energy Vehicle Intelligent Control and Simulation Test Technology, Chengdu, China; 3 Sichuan Key Laboratory of Vehicle Measurement, Control and Safety, Chengdu, China; 4 Huai’an Research Center of Applied Technology, Xihua University, Huai’an, China; Sunway University, MALAYSIA

## Abstract

Lithium-ion batteries are widely used in electric vehicles and portable electronic devices. Accurate estimation of the State of Health (SOH) is essential to guarantee their safe and reliable operation. Electrochemical Impedance Spectroscopy (EIS) can characterize the internal electrochemical aging properties of batteries. However, traditional EIS-based methods only adopt single impedance parameters, which fail to fully describe the coupled aging behaviors and thus suffer from unsatisfactory prediction accuracy. To address this issue, this paper proposes a lithium-ion battery SOH prediction method combining multi-feature combinations of EIS and optimized Back Propagation (BP) neural network. Firstly, we analyze the cyclic aging experimental data of the same type of batteries under different operating conditions. Spearman’s Rank Correlation Coefficient (SRCC) is employed to select valid features highly correlated with capacity degradation, and two sets of EIS multi-feature combinations are established for comparative analysis. Secondly, three optimization algorithms, namely Particle Swarm Optimization (PSO), Genetic Algorithm (GA) and Ant Colony Optimization (ACO), are used to optimize the initial parameters of the BP neural network, which overcomes the drawback that the conventional BP model is prone to falling into local optima. The experimental results reveal that under the operating conditions of 35C01 and 35C02 with insufficient samples and prominent data noise, the BP neural networks optimized by ACO and GA achieve superior prediction performance on the test set, with lower Mean Absolute Error (MAE), Root Mean Squared Error (RMSE) and Mean Absolute Percentage Error (MAPE). For 45C01 and 45C02 with sufficient samples and low data noise, ACO-BP and GA-BP maintain stable prediction performance. The proposed method realizes the organic integration of electrochemical mechanism analysis and data-driven modeling, and effectively improves the prediction robustness and generalization ability. It provides a high-precision and practical technical solution for the online SOH monitoring of lithium-ion batteries.

## 1 Introduction

Lithium-ion batteries have shown broad application prospects across multiple fields due to their high energy density, long lifespan, and environmental friendliness. With the growing demand for Electric Vehicles (EVs) and portable electronic devices, the development of lithium-ion batteries continues to advance rapidly [1]. As a crucial component of EVs, batteries significantly influence the driving range. However, as the battery ages, its capacity gradually declines, making accurate monitoring of the battery’s lifespan increasingly important. The SOH of a lithium-ion battery is a key parameter that indicates its health condition or performance status. Since battery aging is a complex process, quantifiable indicators are needed to describe the degradation accurately. In practical applications, different scenarios define SOH in various ways. As the battery ages, its capacity, internal resistance, power, and cycle life change accordingly [2], and SOH can be defined using different features such as capacity, internal resistance, power, and remaining cycle life [3,4]. Currently, definitions based on internal resistance, power, and remaining cycle life are less frequently used, with most applications adopting capacity-based SOH definitions [5]. It is generally accepted that when the battery’s available capacity falls below 80% of its nominal capacity, it is considered to have reached the end of its life.

Numerous studies have been conducted on the SOH of lithium-ion batteries, with mainstream estimation methods including direct measurement, model-based methods, differential analysis, and data-driven approaches [6]. The direct measurement method involves fully charging and then fully discharging the battery under controlled conditions, recording the discharge current and time, and calculating the available capacity using the ampere-hour integral method. Although this method provides highly accurate SOH estimates, it is time-consuming and requires stringent charging/discharging protocols, making it suitable only for offline calibration or validating the accuracy of other SOH estimation methods [5].

The model-based SOH estimation method involves constructing a battery model and using simulation studies to estimate SOH. Common models include the Equivalent Circuit Model (ECM) and the Electrochemical Model (EM) [7–9]. The ECM, a semi-empirical model, uses equivalent circuit elements and parameter identification algorithms, such as least squares, Kalman filters, and particle filters, to simulate the battery’s charging/discharging characteristics and estimate SOH [10]. Typical ECMs range from first-order to high-order RC networks, and researchers have verified that the second-order RC model with a constant phase element (CPE) achieves the best balance between accuracy and complexity [11]. Westerhoff et al. systematically compared 1–5 order RC and fractional-order models and quantified the impacts of State of Charge (SOC) and temperature on key parameters such as ohmic resistance and charge-transfer resistance, laying a standardized foundation for EIS-based ECM analysis [11]. In recent years, automated ECM methods have been developed to reduce manual intervention; Niri et al. proposed a rapid non-invasive SOH estimation scheme using only medium–high frequency EIS data, which shortened detection time to within 5 minutes and maintained an error below 3% [12]. The accuracy of ECM-based methods depends heavily on model structure, and simplified RC circuits inevitably compromise precision, while high-order models improve accuracy at the cost of computational complexity. In contrast, the EM employs partial differential equations to describe internal electrochemical reactions, offering higher accuracy for SOH estimation but requiring substantial computational resources, which limits its practical applicability.

To overcome the parameter coupling limitation of conventional ECMs, the Distribution of Relaxation Times (DRT) method has been widely studied for its strong decoupling ability without relying on predefined circuit structures. Akram et al. adopted regularized DRT to separate SEI, charge-transfer, and diffusion processes, and combined DRT features with long short-term memory (LSTM) to realize high-precision SOH estimation, reducing the error to below 1.5% [13]. The DRT-based method can distinguish different aging mechanisms and significantly improve estimation robustness under varying SOC conditions [13].

Data-driven methods derive features from battery performance data to estimate SOH using machine learning techniques such as Artificial Neural Networks (ANNs), Support Vector Machines (SVMs), fuzzy logic, and Gaussian processes [14]. These methods establish a nonlinear mapping between external health features and SOH by exploiting large datasets, but they demand significant computational power, making them less feasible for small-scale battery systems with limited hardware capabilities [15]. As a typical representative of ANNs, the BP network has been gradually applied in the field of battery SOH prediction in recent studies for its unique advantages: it is excellent at processing high-dimensional nonlinear data, can accurately mine the complex nonlinear correlation between multi-dimensional aging features and SOH through the nonlinear transformation of hidden layer neurons, and has a simple training process, fast convergence speed, and flexible adjustable model complexity, which can be lightweight designed according to the actual hardware conditions of BMS and is more suitable for engineering deployment compared with complex deep learning models.

To further improve the accuracy and generalization of data-driven models, advanced machine learning frameworks have been explored. Tonima et al. extracted multi-dimensional EIS features including impedance values, phase angles, and ECM parameters, and applied the extreme Gradient Boosting (XGBoost) algorithm with Minimum Redundancy Maximum Relevance (mRMR) feature selection to achieve lightweight and high-precision SOH estimation, with R^2^ reaching 0.985 and MAE below 2% [16]. For time-series-dependent state estimation, Bayesian optimization has been used to automate hyperparameter tuning. Wang et al. proposed a Bayesian optimized bidirectional long short-term memory (BO-BiLSTM) model, which effectively avoided overfitting and low efficiency caused by manual parameter tuning, significantly improving SOC estimation accuracy [17].

For vehicle-mounted applications that lack dedicated EIS hardware, virtual EIS technology provides a feasible solution. Khurana et al. reconstructed virtual EIS signals from current and voltage data without extra hardware, and constructed a hybrid prediction framework combining SVR optimized by Cuckoo Search Algorithm (CSA) and Extended Kalman Filter (EKF) filtering, which reduced SOH error to below 2.5% and improved accuracy by 30% compared with traditional EKF [18].

In addition to the preceding methods, Electrochemical Impedance Spectroscopy (EIS) has emerged as a viable tool for SOH determination and has become a research hotspot in combination with data-driven models in recent years. EIS is a non-destructive testing approach that provides rich information by applying a modest Alternating Current (AC) or voltage perturbation to the battery and measuring the related voltage or current response. The test results yield impedance data at different frequencies, which can be shown as Nyquist plots, illustrating the real part and imaginary part of the impedance. The EIS results at various frequency ranges correspond to the primary electrochemical reactions occurring inside the battery (such as SEI film growth, charge transfer process, and lithium ion diffusion process), which are the essential internal factors leading to capacity deterioration. Traditional EIS-based SOH research only extracts a small number of single impedance parameters (such as ohmic internal resistance, charge transfer resistance) to characterize battery aging, which can only reflect a single aging process and leads to insufficient feature information and low prediction accuracy due to the coupling of multiple aging reactions of lithium batteries. Therefore, the extraction of EIS multi-feature combinations has become an important innovative direction to break through the limitations of traditional research: by extracting comprehensive features from EIS Nyquist plots in terms of impedance numerical values, curve shape parameters, and fitting model coefficients, it can fully characterize the multi-coupling aging process of batteries, and the complementary characteristics between multiple features can effectively reduce the interference of complex working conditions such as temperature and SOC on feature sensitivity, improving the robustness and generalization of the feature set.

Combining EIS multi-feature combinations with the BP network has become a novel and effective technical route for lithium battery SOH prediction in recent research. The EIS multi-feature set, which reflects the intrinsic micro electrochemical aging mechanism of batteries, provides high-quality, interpretable input features for the BP network, making up for the defect that traditional data-driven models use macro features and are disconnected from the aging essence; at the same time, the BP network’s strong fitting ability for high-dimensional nonlinear data can fully exploit the information value of EIS multi-features, establish an accurate nonlinear mapping between electrochemical aging characteristics and SOH, and make up for the deficiency that traditional machine learning models such as Support Vector Regression (SVR) have insufficient fitting ability for high-dimensional EIS features. This combination realizes the organic integration of electrochemical mechanism characterization and data-driven prediction, which not only has the strong interpretability of mechanism analysis but also has the high precision of data-driven models, and effectively balances the prediction accuracy and engineering applicability, providing a new feasible method for the accurate on-line monitoring of lithium battery SOH.

## 2. Selection and identification of health features

### 2.1 Electrochemical impedance spectroscopy

The EIS is a powerful characterization technique used to study the dynamics and interfacial processes in electrochemical systems. It operates by applying a small AC voltage perturbation and monitoring the subsequent current response to analyze the impedance characteristics of the system across multiple frequencies [19,20]. This technique is particularly appropriate for studying batteries, fuel cells, electrolytic cells, corrosion research, and other electrochemical devices. During EIS testing, an AC voltage is delivered to the electrochemical system, and the response is measured as the current. The impedance of the system, defined as the ratio of the applied voltage to the resulting current, can be expressed as follows in Equations(1) to (3) [21,22]:


$$\begin{array}{c} V\left(t\right)=V_{\text{0}}\cdot sin(\omega \cdot t) \end{array}$$ (1)

$$\begin{array}{c} I\left(t\right)=I_{\text{0}}\cdot sin(\omega \cdot t+\varphi ) \end{array}$$ (2)

$$\begin{array}{c} Z\left(\omega \right)=\frac{V\left(\omega \right)}{I\left(\omega \right)}=Z_{\text{0}}\cdot \left(cos\left(\varphi )+j\cdot sin(\varphi \right)\right) \end{array}$$ (3)

Where V(t) is the applied voltage, $V_{\text{0}}$ is the voltage amplitude, ω is the angular frequency, I(t) is the current response, φ is the phase angle, $Z_{\text{0}}$ is the impedance magnitude, and j is the imaginary unit. Equation (3) indicates that impedance is a complex number, comprising of real *Z’* and imaginary *Z”* components. By measuring the impedance at different frequencies, an impedance spectrum may be created, which is typically represented in a Nyquist plot or Bode plot.

**Fig 1:**
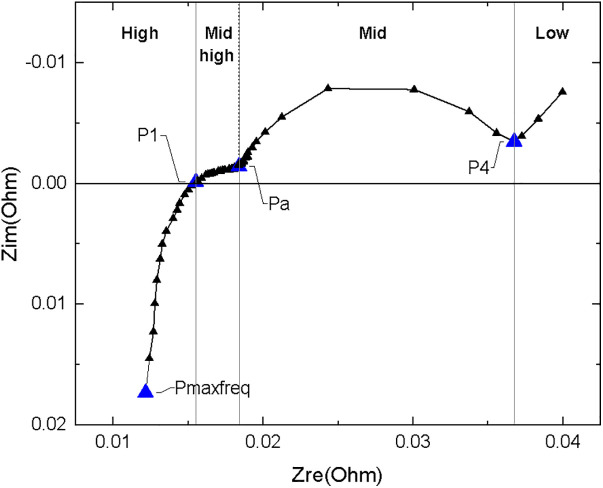
Nyquist chart of electrochemical impedance spectrum.

**Fig 2:**
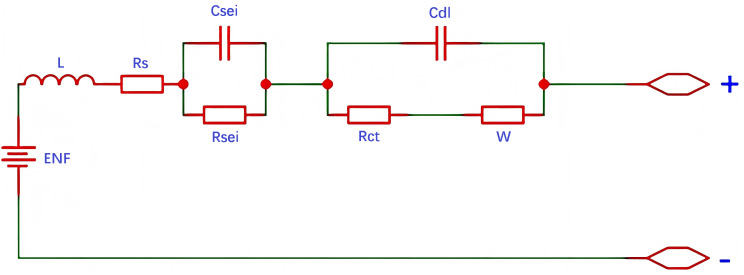
Equivalent circuit.

The Nyquist plot presents the real part of impedance on the horizontal axis and the imaginary part on the vertical axis, offering a comprehensive depiction of the electrochemical impedance in the complex plane. Each point on the Nyquist plot corresponds to a particular frequency, with high frequencies located on the left and low frequencies on the right. When conducting EIS testing on a battery, the test results can be separated into four primary components, as indicated in Fig 1. The corresponding equivalent circuit model based on this Nyquist plot is illustrated in Fig 2.

These four segments are separated into high-frequency (7812.52 Hz – 755.312 Hz), mid-high-frequency (755.312 Hz – 32.425 Hz), mid-frequency (32.425 Hz – 0.1192 Hz), and low-frequency (0.1192 Hz – 0.0149 Hz) ranges. The frequency partitioning reflects the electrochemical processes occurring within the lithium-ion battery. The high-frequency segment represents the inductance behavior due to the electron flow through the wires and the internal electrode winding of the lithium battery, which can be represented by the equivalent circuit element L[17]. The intersection of the Nyquist curve with the real axis corresponds to the ohmic resistance $R_{\Omega }$ or $R_{S}$, representing the internal resistance of the battery, comprising ion and electron routes through the electrolyte and active material pores.

The mid-high-frequency region often displays a tiny semicircle or arc, primarily attributed to the diffusion of lithium ions through the SEI(Solid Electrolyte Interphase) layer. The SEI growth occurs from the irreversible decomposition of the electrolyte, and this part of the impedance can be represented by the parallel combination of SEI Resistance $R_{SEI}$ and SEI Capacitance $C_{SEI}$. The mid-frequency segment is typically characterized by a larger semicircle, related to the charge transfer process at the electrode/electrolyte interface, which can be modeled by the parallel combination of Charge Transfer Resistance $R_{CT}\;$and Double-Layer Capacitance $C_{DL}$[11,23,24]. The low-frequency region, generally depicted as a 45° diagonal line, corresponds to the diffusion of lithium ions in the electrode interface and active materials, usually represented by the Warburg Impedance W.

### 2.2 Dataset description

The battery aging dataset used in this study was obtained from a public dataset made available by the University of Cambridge and its collaborators as part of their research on using machine learning to identify lithium-ion battery degradation patterns from impedance spectra [21]. The experiment was conducted on 12 commercially available 45 mAh Eunicell LR2032 lithium-ion coin cells, using continuous charge and discharge cycles. The electrolyte material of the batteries was LiCoO₂/graphite. The experiments were performed at three distinct temperatures: 35°C (35C01 and 35C02), and 45°C (45C01 and 45C02). Each cycle involved Constant Current-Constant Voltage charging at a 1C rate (45 mA) up to 4.2 V, followed by constant current discharging at a 2C rate (90 mA) to 3 V. The C-rate is a measure of the charging and discharging current relative to the rated capacity. EIS measurements were performed over a frequency range of 0.02 Hz to 20 kHz under different operating conditions at nine different stages (according to SOC, with or without direct current and rest, denoted as stages I to IX). The battery capacity was measured after every odd-numbered cycle. This analysis utilizes EIS and capacity data obtained from these cycles.

**Fig 3:**
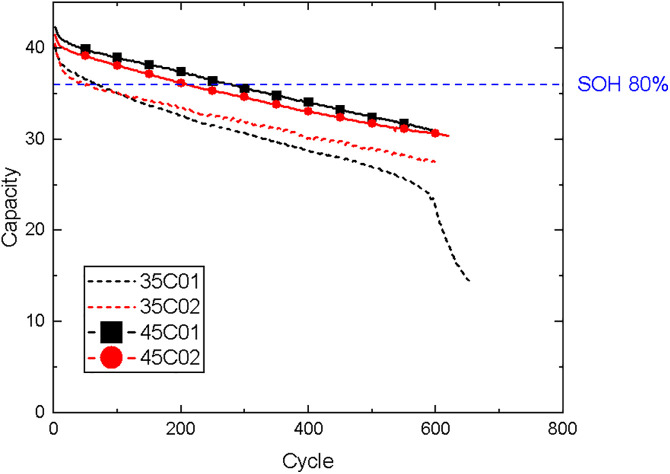
Capacity attenuation curve of 4 batteries.

It is generally acknowledged that when the available capacity of a battery falls below 80% of its nominal capacity, it is regarded to have reached the end of its life. Therefore, assessing the relationship between battery capacity and EIS test findings before the battery life ends is essential. Fig 3 illustrates the capacity deterioration curves of the four batteries. It was observed that, at 25°C, there were relatively few data points where the SOH of the eight batteries stayed above 80%, indicating that the cyclic aging studies at this temperature were insufficient for providing effective lifespan prediction information. Additionally, the performance variations toward the end of battery life could not be fully represented. Therefore, this study selected the data from the battery groups 35C01, 35C02, 45C01, and 45C02 for further research.

### 2.3 Feature Selection

**Fig 4:**
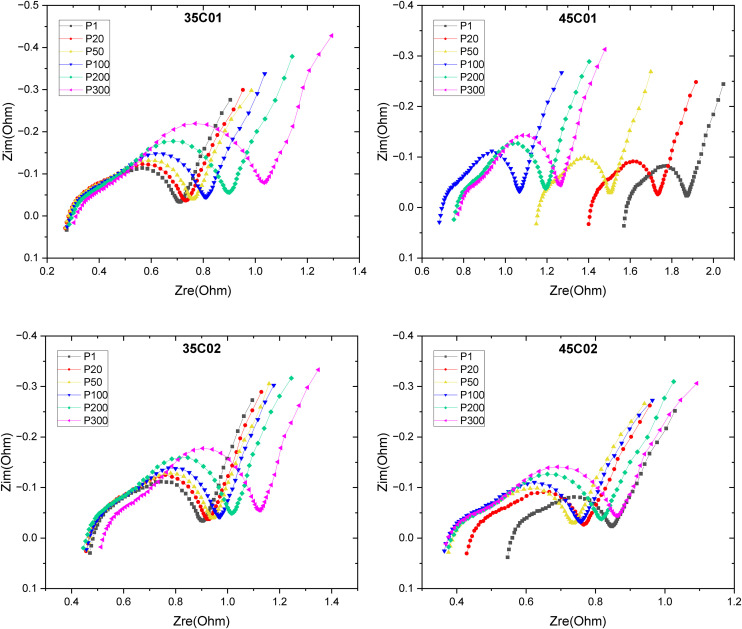
EIS test results of four groups of data under different cycles.

To investigate the relationship between battery capacity degradation and EIS test results at the given condition, the EIS test results of four selected battery groups were sampled at the 1st, 20th, 50th, 100th, 200th, and 300th cycles, totaling six different cycle counts. The plotted EIS test results are shown in Fig 4. It was observed that the data from the 45C01 group exhibited significant variations compared to the other three groups, mainly in the position of the EIS curve along the horizontal axis. The EIS curves of the 35C01, 35C02, and 45C02 groups remained relatively fixed along the real-axis coordinate, whereas the EIS curve of the 45C01 group showed a gradual rightward shift as the cycle count increased, stabilizing around the 100th cycle. The EIS curve of the 45C01 group showed trends similar to those of the other three groups beyond the 100th cycle, suggesting that external factors might have contributed to the discrepancy observed in the first 100 cycles.

**Fig 5:**
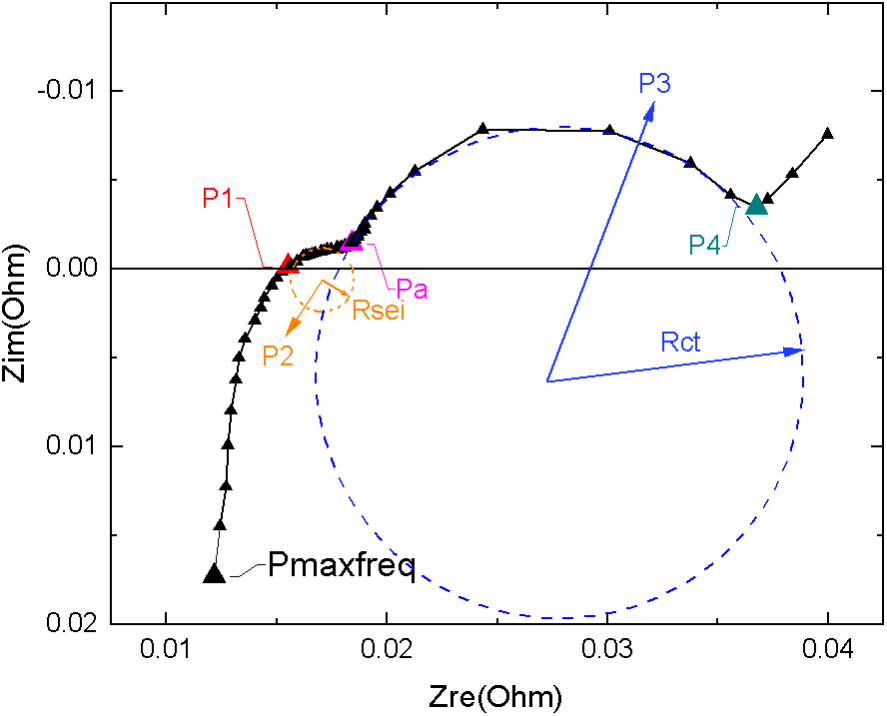
Use the EIS curve to find the characteristics related to battery life.

The EIS test results showed an overall increase in ohmic resistance (indicated by the rightward shift of the intersection between the EIS curve and the real axis), relatively minor changes in SEI film resistance, and significant increases in charge transfer resistance and diffusion impedance. Therefore, ohmic resistance, SEI film resistance, charge transfer resistance, diffusion impedance, and their associated parameters were selected as features to assess battery aging. Taking the EIS test results of the last cycle in group 35C02 as an example, these candidate features are summarized in Fig 5, where the leftmost point of the curve corresponds to the highest frequency point in the EIS test.

Notably, the position of this point does not exhibit a linear relationship with the cycle count, especially in the data of group 45C01, where the entire EIS curve shows an irregular horizontal shift. This suggests that the correlation between the curve position and the SOH is lower than that between the curve shape and SOH. To extract more shape – related features, the horizontal coordinate of this point is labeled as “P_maxfreq_x_,” and subsequent features (such as intercepts of fitted lines, centers of fitted circles, etc.) will be adjusted relative to “P_maxfreq_x_” to enhance their correlation with the battery’s SOH.

In this study, a total of seven potential features related to battery capacity degradation were extracted as shown in Fig 5, as follows:

Physical features are derived from EIS curve fitting using the equivalent circuit model. With clear electrochemical implications, they characterize the internal operating mechanisms of batteries. This category includes:

Rs’: Ohmic resistance (the intersection of the EIS curve with the real – axis and the intersection with the yellow line), the difference between P_1_x_ and “P_maxfreq_x_”;

Rsei: SEI film resistance, corresponding to the radius of the arc fitted by the orange line segment;

Rct: Charge – transfer resistance, corresponding to the radius of the arc fitted by the blue line segment;

Geometric features are directly extracted from the Nyquist plot to describe the morphology of impedance curves, covering coordinate relationships and extreme values of the EIS profile. This category includes:

Rct_x’: The difference between the P_3_x_ coordinate of the center of the charge – transfer impedance circle and “P_maxfreq_x_”;

∆x: The difference between P_3_x_ and P_2_x_ of the centers of the two circles;

∆y: The difference between P_3_y_ and P_2_y_ of the centers of the two circles;

ymax: The y – coordinate of P_maxfreq._

Symbol Explanation: “##_x/##_y” stands for the x-axis or y-axis value corresponding to the relevant variable.

### 2.4 Feature Correlation Analysis

The SRCC was used to analyze the relationship between these 7 pre-selected features and battery aging.

Theρ(SRCC) is commonly used to measure the strength and direction of the association between two variables. The formula for calculating ρ is as follows:


$$\begin{array}{c} \rho =1-\frac{6\cdot \sum d_{i}^{\text{2}}}{n\cdot (n^{\text{2}}-1)} \end{array}$$ (4)

where $d_{i}$is the difference between the rank values of the two variables for the i-th data point, and n is the total number of observations.

Generally, when |ρ| falls between 0.7 and 1, there is a very strong monotonic relationship between the two variables. If |ρ| is between 0.4 and 0.7, it indicates a moderate correlation; between 0.1 and 0.4 suggests a weak correlation, and values below 0.1 imply no significant correlation.

**Fig 6:**
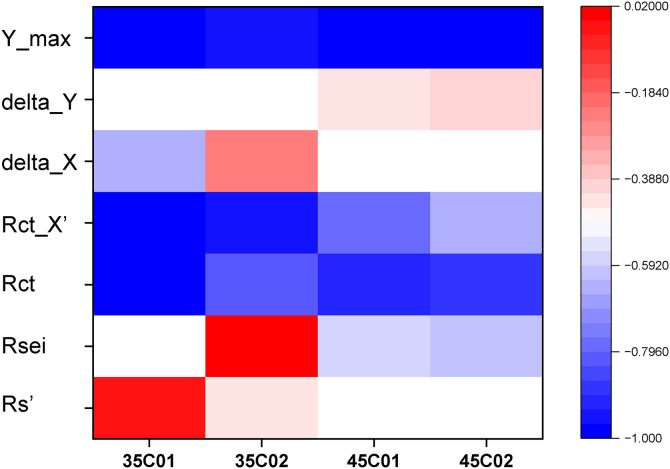
The Spearman rank correlation coefficients of the 7 features.

By processing the data from the public dataset using MATLAB, the Spearman rank correlation coefficients of the 7 features were analyzed, and the results are shown in Fig 6.

### 2.5 Feature selection schemes

To train the SOH estimation model, the features were divided into three different schemes based on the results of the Spearman correlation analysis to comprehensively evaluate the impact of feature selection on model performance:

**Scheme 1**: All features are included, regardless of their correlation coefficients. This scheme aims to ensure the model’s generalization ability by incorporating as much information as possible, even if some features have low correlations. In complex nonlinear models, low-correlation features might provide additional information to improve model performance.

**Scheme 2**: Features with absolute correlation values above 0.9 are selected, including $R_{ct\;}and\;y_{max}$. These features have a high correlation with capacity changes across different groups, indicating their significant impact on SOH estimation.

## 3. Algorithm models and evaluation metrics

### 3.1 Backpropagation neural network estimation model

**Fig 7:**
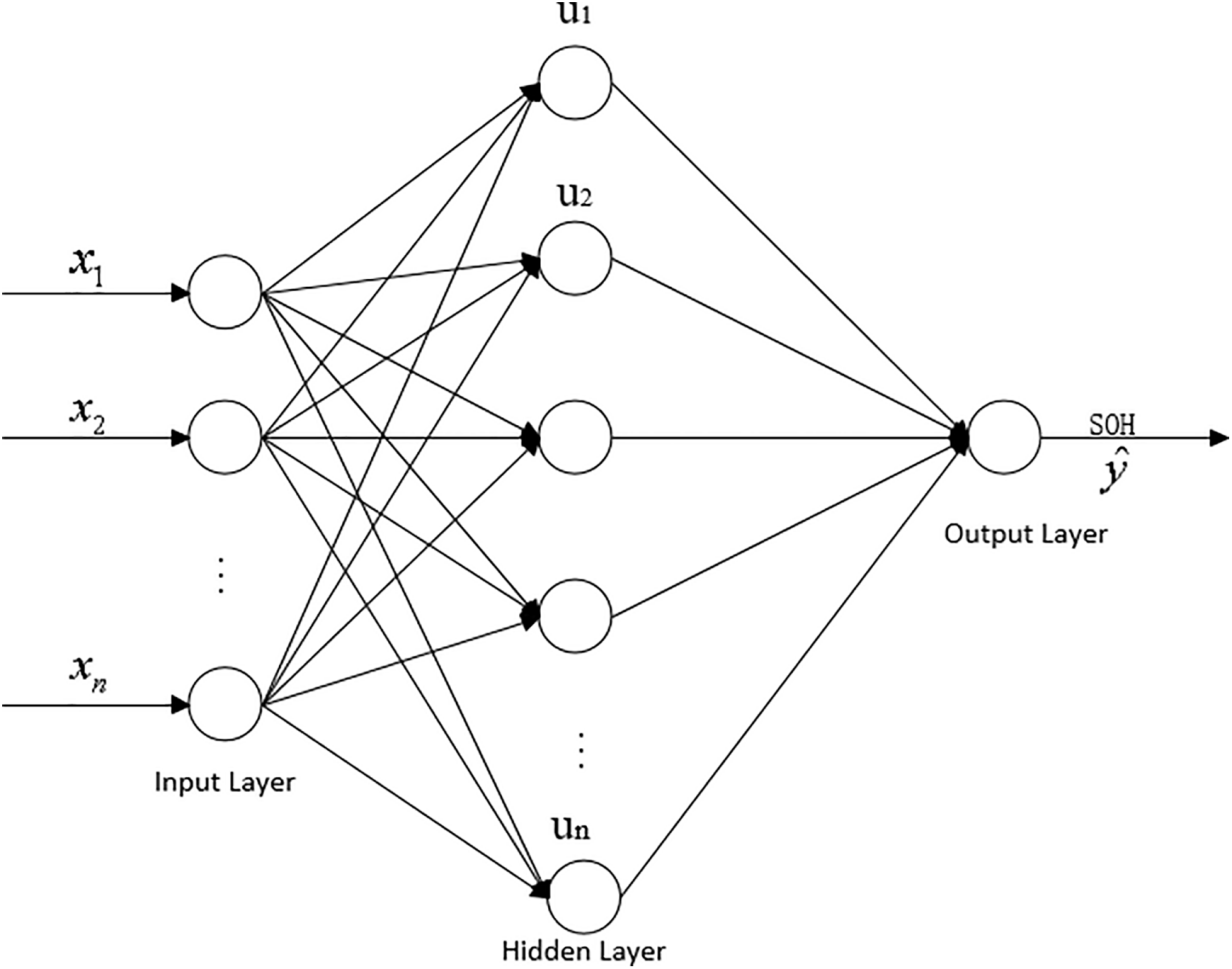
Classical BP neural network structure.

BP is a multilayer feedforward neural network model, primarily composed of input and output nodes, along with several hidden layers, where connections between layers are characterized by weights. By adjusting these weights, the network learns to map relationships between inputs and outputs. The core mechanism of this model lies in optimizing network parameters through minimization of a loss function to address nonlinear regression problems, with its architecture illustrated in Fig 7.

This study establishes a three-layer BP neural network prediction model with a classic architecture. The structural parameters of each layer and relevant training strategies are described in detail as follows:

#### 1) Network structure and parameter configuration.

Input layer: The number of neurons is determined by the dimension of sample features. This layer receives standardized feature vectors without adopting any activation function.

Hidden layer: A single hidden layer containing 10 neurons is configured. The tansig function is selected as the activation function, which endows the model with strong nonlinear fitting capability to capture complex correlations within datasets.

Output layer: One neuron is arranged to adapt to the regression prediction task. The purelin function is employed as the activation function, which normalizes the outputs to the range of (0, 1) and conforms to the numerical characteristics of the State of Health (SOH) of batteries.

#### 2) Model training and optimization strategy.

Mean Squared Error (MSE) is used as the loss function to calculate the error between predicted values and actual values. The traditional BP algorithm suffers from drawbacks such as easy trapping in local optima and poor convergence stability. To address these problems, Genetic Algorithm (GA), Ant Colony Optimization (ACO) and Particle Swarm Optimization (PSO) are applied to optimize the learning rate and regularization coefficient of the network. Three hybrid models, namely GA-BP, ACO-BP and PSO-BP, are constructed accordingly. This hybrid strategy balances global search ability and local convergence accuracy, and effectively improves the generalization performance and prediction stability of the model.

To eliminate the interference caused by manual parameter tuning, the remaining parameters adopt the default values of MATLAB. In addition, the population size and maximum iteration number are set uniformly for the three metaheuristic algorithms to ensure fair and reliable comparative experiments. The detailed parameter settings are presented in Table 1.

**Table 1:** Parameter settings of GA, PSO, and ACO algorithms.

Algorithm	Parameter	Value
GA	Population size	10
	Maximum generations	10
	Crossover probability	0.8
	Mutation probability	0.2
PSO	Particle number	10
	Maximum iterations	10
	Inertia weight *w*	0.7
	Learning factors *c*, *c*	1.5, 1.5
ACO	Ant number	10
	Maximum iterations	10
	Pheromone factor α	1.0
	Heuristic factor β	1.0
	Pheromone evaporation rate ρ	0.5

### 3.2 BP neural network model optimized by genetic algorithm

The GA is a global optimization algorithm proposed based on the laws of natural evolution and genetic mechanisms. This method explores the optimal solution by simulating the evolutionary process of living organisms. When this method is applied to BP neural networks, it can adjust the weights and biases in the BP neural network with the help of GA’s global search function, thereby preventing the BP model from getting stuck in a local optimal state and improving the convergence speed and accuracy of the network [25].

The main idea of the genetic algorithm is to simulate the evolution process of organisms and select the best solution through natural means. When using this method to optimize the backpropagation neural network, the parameters of the network are generally set in the form of chromosomes, where each gene represents the specific value of a certain parameter. By continuously updating the chromosomes of the population, that is, continuously updating the parameters of the neural network, better regression prediction results are expected.

**Fig 8:**
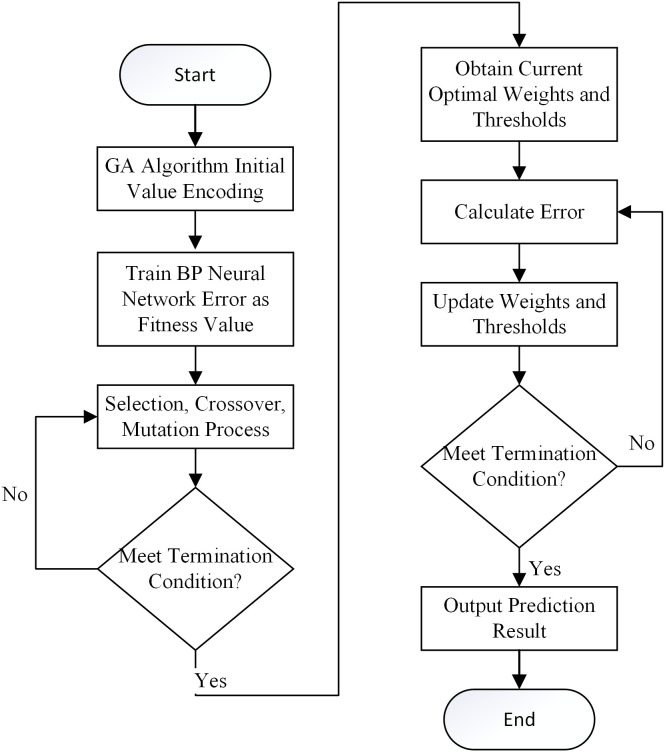
GA flowchart.

The core process of the genetic algorithm is to first create an initial population, that is, randomly select a certain number of individuals as the initial population. Each individual represents a possible solution to the problem, usually displayed in the form of binary strings, real number vectors or other encoding forms. Population Size is also an important parameter, which affects the search ability and convergence speed of the algorithm. Then, it is necessary to evaluate the fitness. Here, according to the objective function of the problem, the fitness value of each individual is calculated. The level of this value can positively reflect the excellence of the individual, and the closer it is to the optimal solution of the problem. The design of the fitness function is the key to the genetic algorithm and will directly affect the performance of the algorithm.The main steps of GA are illustrated in Fig 8.

### 3.3 BP neural network model optimized by ant colony optimization algorithm

**Fig 9:**
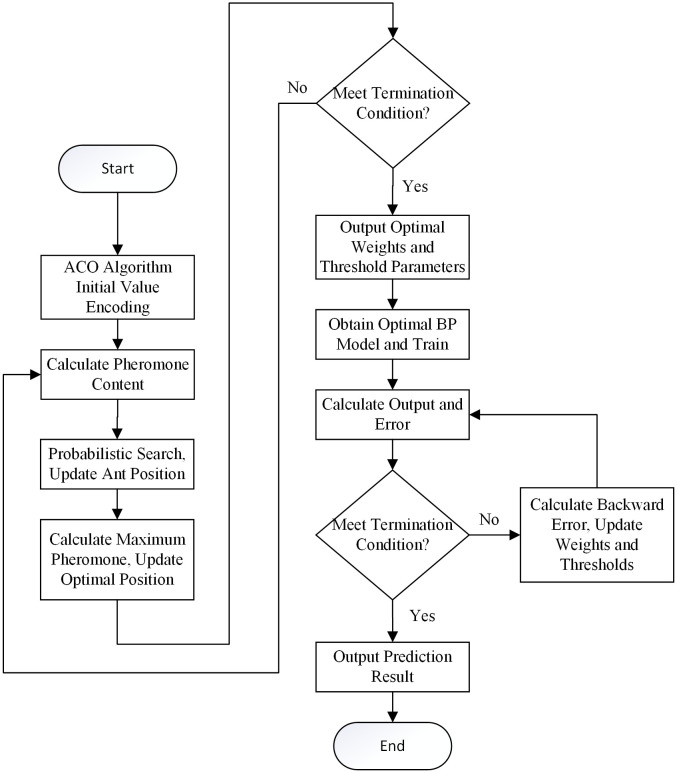
ACO algorithm chart.

ACO is an optimization technology based on swarm intelligence, inspired by the foraging behavior of ant colonies. Its concept originates from the study of the process by which ants search for food. The basic idea of this method is to imitate the action of ants spreading pheromones when exploring food, thereby gradually finding the optimal path. When ants are on a path, they release pheromones, and other ants select paths based on the amount of pheromones; paths with more pheromones have a higher probability of being chosen. Over time, pheromones evaporate, and the pheromone concentration on excellent paths gradually increases, thereby guiding the ant colony to find the optimal solution. Combining ACO with backpropagation neural networks can improve the training efficiency and performance of neural networks [26].The main steps of ACO are illustrated in Fig 9.

### 3.4 BP neural network model optimized by particle swarm optimization algorithm

The PSO algorithm is inspired by the foraging behaviors of bird flocks and fish schools. The core concept of this algorithm is to simulate the movement of particles in the solution – space to find the optimal solution. Each particle represents a possible solution and can adjust its position and velocity based on individual and group experiences.

**Fig 10:**
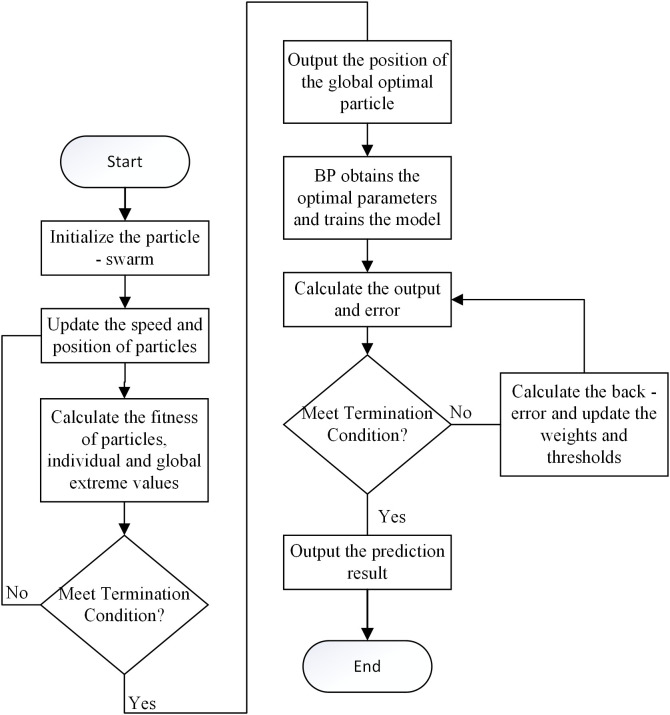
PSO flowchart.

Optimizing the BP neural network using the PSO algorithm specifically involves leveraging the global search ability of PSO to optimize the weight and bias parameters of the BP neural network. This helps prevent the BP algorithm from getting trapped in local optimal solutions and improves the convergence speed and accuracy of the network [27]. The main steps of PSO are illustrated in Fig 10.

### 3.5 Evaluation metrics

The model’s predictive accuracy is evaluated using the following three metrics: MAE, RMSE, and MAPE, whose detailed computational formulas are provided below. These metrics collectively assess the model’s predictive performance and stability, enabling the selection of the optimal model for the SOH estimation.


$$\begin{array}{c} MAE=\frac{\text{1}}{n}\sum_{i=1}^{n}\left|y_{i}-\hat{y_{i}}\right| \end{array}$$ (5)

$$\begin{array}{c} RMSE=\sqrt{\frac{\text{1}}{n}\sum_{i=1}^{n}(y_{i}-{\hat{y}}_{i}{)}^{\text{2}}} \end{array}$$ (6)

$$\begin{array}{c} MAPE=\frac{\sum_{i=1}^{n}\left|\frac{y_{i}-{\hat{y}}_{i}}{{\hat{y}}_{i}}\right|}{n}\times 100\% \end{array}$$ (7)

where $y_{i}\;$represents the actual values, ${\hat{y}}_{i}$are the predicted values. MAE reflects the average prediction error, providing a direct measure of prediction accuracy, while RMSE emphasizes larger errors, offering a stricter evaluation of model performance. MAPE expresses results as percentages and is less influenced by extreme values, allowing for comprehensive comparisons across datasets of different scales. The combined use of these three evaluation metrics offers a more holistic and multi-dimensional assessment of model performance, facilitating a deeper understanding of the model’s strengths and limitations while mitigating the constraints of relying on a single metric.

## 4 Experimental results and analysis

To validate the effectiveness of the extracted feature combinations, one group of data from the previously mentioned public dataset at 35°C and another group at 45°C were selected as the training and testing sets to evaluate the model’s accuracy. Due to significant discrepancies in the EIS test results of the 45C01 group during the first 100 cycles, the training set was established using the data from 35C01 and 45C02 to ensure a sufficient sample size. Meanwhile, the data from 35C02 and 45C01 were selected as the testing set after removing the results from the first 100 cycles. The estimated values of the three optimized models were then compared with reference values to identify the most accurate predictive model.

**Fig 11:**
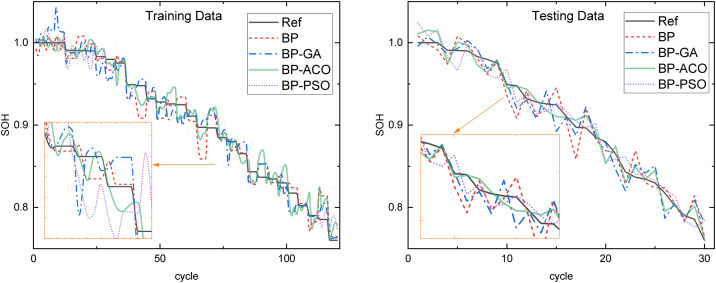
Performance comparison under different estimation models.

**Fig 12:**
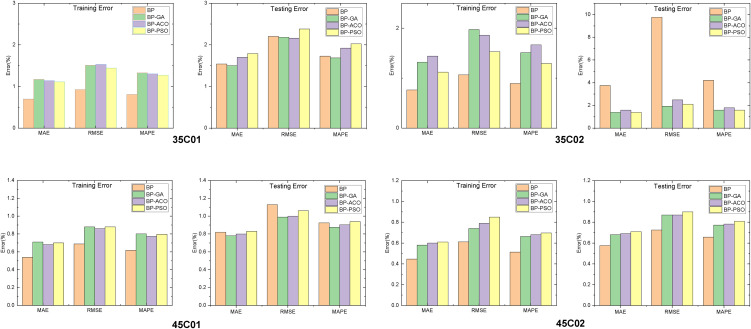
Error between SOH estimates and true values under different estimation models.

Fig 11 and Fig 12 present the differences in various error evaluation metrics of BP neural networks optimized by different algorithms under Scheme 2. Analysis of the graphs indicates that the BP neural networks optimized by GA and ACO deliver remarkably better prediction performance than the PSO-optimized model and the original unoptimized BP model. Overall, the ACO-BP and GA-BP models achieve high accuracy for SOH prediction, with lower prediction errors and the optimal stability among all tested models.

## 5 Conclusion

This study proposes a novel method for estimating the SOH of lithium-ion batteries by integrating multi-feature fusion of EIS and BP neural networks. This approach addresses the drawbacks of conventional EIS-based methods, which rely solely on single impedance parameters and fail to fully characterize the coupled aging behaviors of batteries.

Different from traditional data-driven models that adopt simple macroscopic features and lack interpretability in terms of electrochemical mechanisms, the proposed method extracts comprehensive aging information from EIS data to reflect the microscopic aging characteristics inside batteries. It realizes the effective combination of electrochemical mechanism analysis and data-driven prediction. In this research, seven typical EIS features are extracted, and the SRCC is used to quantitatively select valid features highly correlated with battery capacity degradation. Four different EIS multi-feature combination schemes are designed and compared. The results demonstrate that Scheme 2 has the best feature complementarity, which effectively improves the accuracy of model training and testing. To overcome the problem that conventional BP neural networks are prone to falling into local optima, three classic intelligent optimization algorithms, GA, ACO and PSO, are applied to optimize the initial parameters of the BP model.

Experimental results demonstrate that under the working conditions of 35C01 and 35C02 with insufficient samples and high data noise, the BP networks optimized by GA and ACO achieve better prediction performance with lower MAE, RMSE and MAPE. For 45C01 and 45C02 with adequate samples and low data noise, the two optimized models maintain stable estimation performance. Overall, the BP models optimized by ACO and GA outperform other comparative models in comprehensive performance.

Under the experimental conditions in this work, the proposed method achieves favorable estimation accuracy for battery aging data. The established model significantly improves the accuracy and stability of lithium-ion battery SOH estimation, and possesses great engineering application potential for electric vehicles and energy storage systems. Nevertheless, the adaptability of the model in complex and variable practical working conditions still needs further investigation. Future research will conduct validation using more diverse battery datasets to further optimize the method and enhance its generalization ability and universality.

## Supporting information

S1 File Raw EIS dataset of cell 35C01. Raw EIS test data matrix of lithium battery cell 35C01 under aging cycles.
(ZIP)

S2 File Multi-feature dataset of cell 35C01. Extracted multi-feature matrix from the raw EIS data of cell 35C01.
(ZIP)

S3 File Raw EIS dataset of cell 35C02. MATLAB matrix file storing the raw electrochemical impedance spectroscopy measurement data of lithium battery cell 35C02 under cyclic aging tests.
(ZIP)

S4 File Multi-feature extracted dataset of cell 35C02. MATLAB matrix file containing the multi-dimensional EIS characteristic parameters extracted from the raw impedance data of cell 35C02, used as the input feature set of prediction models.
(ZIP)

S5 File Raw EIS dataset of cell 45C01. Raw electrochemical impedance spectrum data of lithium battery cell 45C01.
(ZIP)

S6 File Multi-feature dataset of cell 45C01. Extracted EIS multi-feature matrix of cell 45C01 for model training.
(ZIP)

S7 File Raw EIS dataset of cell 45C02. Raw aging EIS measurement data matrix of lithium battery cell 45C02.
(ZIP)

S8 File Multi-feature dataset of cell 45C02. Extracted multi-dimensional EIS feature matrix derived from raw test data of cell 45C02.
(ZIP)

S9 File Feature extraction script. General MATLAB function script to automatically extract multi-dimensional characteristic features from raw EIS impedance curves.
(ZIP)

S10 File BP model code. MATLAB script of the original back propagation neural network benchmark model for battery SOH prediction without intelligent optimization algorithm.
(ZIP)

S11 File ACO-BP model code. MATLAB script for the ant colony ptimization optimized back propagation neural network prediction model, which realizes the SOH prediction of lithium-ion batteries based on EIS multi-feature inputs.
(ZIP)

S12 File GA-BP model code. MATLAB script for genetic algorithm optimized back propagation neural network SOH prediction model.
(ZIP)

S13 File PSO-BP model code. MATLAB script for particle swarm optimization optimized back propagation neural network SOH prediction model.
(ZIP)
