# Supplementary material for: Research on predicting the state of health of lithium-ion batteries via back propagation network based on multi-feature combination of electrochemical impedance spectroscopy
Source: PLoS One. 2026 Aug 6;21(8):e0354706. doi: 10.1371/journal.pone.0354706 (PMC13446704; doi:10.1371/journal.pone.0354706)
Supplement: Supplementary file 1 [file pone.0354706.s015.docx]

**Response to Reviewer #2**

**Dear Reviewer,**

Thank you very much for your careful review and valuable suggestions, which have helped us greatly improve the novelty, clarity, and reproducibility of this manuscript. We have carefully addressed all your comments point by point, and the revisions are marked in red in the revised version.

**Comment 1:** The combination of EIS feature extraction and BP neural networks is already widely used in the literature. This approach is often employed as a baseline method in related studies. The novelty is limited, and the authors have not conducted a comprehensive review of existing EIS-based SOH estimation methods.

**Response:**

We sincerely appreciate the reviewer’s professional and insightful comments on the novelty and literature review. We fully acknowledge that the combination of EIS feature extraction and BP neural network has been applied as a common baseline in battery SOH estimation. However, based on the comprehensive review of the latest representative studies (Refs. [11–16]), our work presents clear and substantial improvements in feature engineering, feature selection strategy, and model optimization mechanism, which effectively enhance the prediction accuracy, interpretability, and stability compared with traditional EIS–BP frameworks.

1. Comprehensive review of existing EIS‑based SOH estimation methods

As summarized in the revised Introduction, mainstream EIS‑driven SOH approaches can be categorized into three branches:

Equivalent circuit model (ECM) parameter‑based methods

Ref. [11] systematically analyzed various ECMs for lithium‑ion batteries using EIS, focusing on circuit topology and parameter identification, but only used single physical parameters (e.g., ohmic resistance, charge‑transfer resistance) for state evaluation, lacking multi‑feature fusion.

Ref. [12] achieved rapid SOH estimation via automated EIS and ECMs, but still relied on conventional circuit fitting parameters without geometric feature mining.

Relaxation time distribution (DRT)‑based methods

Ref. [13] integrated DRT parameters with LSTM network for SOH prediction, which improves time‑series feature learning, but depends on complex DRT calculation and cannot explicitly distinguish physical and geometric characteristics of EIS.

Direct EIS feature + machine learning methods

Refs. [14, 15] adopted machine learning models combined with EIS features for SOH estimation, while mostly using shallow networks or fixed feature sets without targeted optimization for BP local optimum issue.

Ref. [16] proposed Bayesian optimization‑BiLSTM for SOC estimation, which belongs to time‑series sequential modeling, significantly different from our static EIS feature‑based SOH prediction framework.

2. Novelty and improvements of this work

Compared with the above studies, our method breaks through the limitations of traditional EIS–BP framework in the following aspects:

Dual‑type multi‑feature fusion

Instead of single ECM parameters or simple impedance values, we extract physical parameters (from ECM fitting) and geometric features (from Nyquist plot) simultaneously, forming a more comprehensive feature set that reflects both electrochemical mechanism and curve morphology.

SRCC‑driven feature screening

We use Spearman’s rank correlation coefficient to quantitatively measure feature‑degradation correlation and construct optimal feature combinations, avoiding redundant or weakly correlated features that are common in conventional methods.

optimized BP network

After comparing multiple schemes, this study adopts Ant Colony Optimization (ACO) and Genetic Algorithm (GA) to optimize the learning rate and regularization coefficient of the BP neural network. This approach addresses the problem that conventional BP neural networks tend to fall into local optima, and the optimized model achieves higher accuracy than the original BP network and the PSO-optimized BP network (PSO-BP).

**Comment 2:** It fails to provide a comprehensive overview of the mainstream directions and methods in current EIS-based SOH estimation research.

**Response:**

We appreciate this suggestion. We have expanded the Introduction to provide a systematic overview of the mainstream directions in EIS-based SOH estimation:

(1) Single-parameter methods using only ohmic resistance or charge transfer resistance;

(2) Equivalent circuit model (ECM)-based methods that fit impedance spectra with circuit elements;

(3) Multi-feature and machine learning-based methods that combine EIS features with data-driven models.

We have also clarified the position of our work in this context, showing that our multi-feature fusion and optimized BP network belong to the third category but with specific improvements in feature design and model optimization.

**Comment 3:** The manuscript confuses physical parameters from equivalent circuit models with geometric features. The proposed features (ohmic resistance, SEI resistance, and charge transfer resistance) are obtained through EIS curve and should be considered geometric features. Although they are related to physical properties, the two concepts should not be used interchangeably.

**Response:**

We thank the reviewer for pointing out this confusion. We have revised the manuscript to clearly distinguish and define the two types of features:

Physical parameters: Obtained by fitting the EIS curve with an equivalent circuit model, including ohmic resistance $\text{R}_{\text{S}}$, SEI film resistance $\text{R}_{\text{SEI}}$, and charge transfer resistance $\text{R}_{\text{CT}}\text{ }$. These parameters have clear physical meanings related to internal electrochemical processes.

Geometric features: Directly extracted from the Nyquist plot, such as the center coordinates and radius of the two arcs, the horizontal difference between arc centers, and the maximum imaginary part value. These features describe the shape of the impedance curve.

We explicitly state that the multi-feature combination in this work integrates both types of features to comprehensively characterize battery aging, and the two terms are no longer used interchangeably.

**Comment 4:** The dataset is extremely small and insufficient. Only two groups of battery data, totaling a few dozen samples, are used as the test set, which cannot support claims of “high accuracy.”

**Response:**

Thank you for this valuable comment. We have supplemented and revised the description of the dataset in the revised manuscript.

In this study, experimental data collected from the same type of lithium-ion batteries under four different operating conditions are adopted. The total number of samples is well over 200, covering the full aging cycle of the batteries from the fresh state to an SOH of 80%. The sufficient sample size reduces errors caused by random data splitting and improves the reliability of experimental results.

In addition, we have revised the overstated expressions in the original text. The relevant statement is updated to: Under the experimental conditions of this work, the proposed method achieves favorable estimation accuracy for battery aging data. We also acknowledge that further validation using datasets from various types of batteries is required in future research, which has been included in our research plan.

**Comment 5:** The method used to extract geometric features is not clearly described, nor is it specified at which SOC levels the EIS data were collected. The model structure and parameters are also insufficiently detailed, including the number of hidden layers, number of neurons, learning rate, regularization, and number of training epochs.

**Response:**

We thank the reviewer for pointing out the missing methodological details, and we have made corresponding supplements and revisions in the revised manuscript:

(1) The structure and parameter settings of the BP neural network have been fully supplemented.

(2) We have added the parameter configurations of three optimization algorithms, including Genetic Algorithm (GA), Particle Swarm Optimization (PSO) and Ant Colony Optimization (ACO).

(3) The data source and experimental test methods refer to Reference [24]: Zhang Y W, Tang Q C, Zhang Y, et al. Identifying degradation patterns of lithium ion batteries from impedance spectroscopy using machine learning. Nature Communications, 11(1): 1706.

(4) The extraction of EIS features follows the method presented in Reference [11]: Westerhoff U, Kurbatov D, Kroger T, Sauer D U. Analysis of lithium-ion battery models based on electrochemical impedance spectroscopy. Energy Technology, 2016, 4(10): 1228-1238. https://doi.org/10.1002/ente.201600154.These additions improve the

reproducibility of our work, and all details are clearly marked in red.

Thank you again for your valuable comments and suggestions, which have significantly improved the quality and clarity of this manuscript.

**Sincerely,**

**Fei Chen**
