# Supplementary material for: Research on predicting the state of health of lithium-ion batteries via back propagation network based on multi-feature combination of electrochemical impedance spectroscopy
Source: PLoS One. 2026 Aug 6;21(8):e0354706. doi: 10.1371/journal.pone.0354706 (PMC13446704; doi:10.1371/journal.pone.0354706)
Supplement: Peer review history

# Peer review: Decision Letter 0

**DOI:** 10.1371/journal.pone.0354706.r001

**Authors:** Shaheer Ansari

20 Apr 2026

PONE-D-26-11604

Research on Predicting the State of Health of Lithium-Ion Batteries via Back Propagation Network Based on Multi-Feature Combination of Electrochemical Impedance Spectroscopy

PLOS One

Dear Dr. fei,

Thank you for submitting your manuscript to PLOS ONE. After careful consideration, we feel that it has merit but does not fully meet PLOS ONE’s publication criteria as it currently stands. Therefore, we invite you to submit a revised version of the manuscript that addresses the points raised during the review process.

Please submit your revised manuscript by Jun 04 2026 11:59PM. If you will need more time than this to complete your revisions, please reply to this message or contact the journal office at plosone@plos.org. When you're ready to submit your revision, log on to https://www.editorialmanager.com/pone/ and select the 'Submissions Needing Revision' folder to locate your manuscript file.

Please include the following items when submitting your revised manuscript:

- A letter that responds to each point raised by the academic editor and reviewer(s). You should upload this letter as a separate file labeled 'Response to Reviewers'.
- A marked-up copy of your manuscript that highlights changes made to the original version. You should upload this as a separate file labeled 'Revised Manuscript with Track Changes'.
- An unmarked version of your revised paper without tracked changes. You should upload this as a separate file labeled 'Manuscript'.

If you would like to make changes to your financial disclosure, please include your updated statement in your cover letter. Guidelines for resubmitting your figure files are available below the reviewer comments at the end of this letter.

If applicable, we recommend that you deposit your laboratory protocols in protocols.io to enhance the reproducibility of your results. Protocols.io assigns your protocol its own identifier (DOI) so that it can be cited independently in the future. For instructions see: https://journals.plos.org/plosone/s/submission-guidelines#loc-laboratory-protocols. Additionally, PLOS ONE offers an option for publishing peer-reviewed Lab Protocol articles, which describe protocols hosted on protocols.io. Read more information on sharing protocols at https://plos.org/protocols?utm_medium=editorial-email&utm_source=authorletters&utm_campaign=protocols.

As the corresponding author, your ORCID iD is verified in the submission system and will appear in the published article. PLOS supports the use of ORCID, and we encourage all coauthors to register for an ORCID iD and use it as well. Please encourage your coauthors to verify their ORCID iD within the submission system before final acceptance, as unverified ORCID iDs will not appear in the published article. *Only*  the individual author can complete the verification step; PLOS staff *cannot*  verify ORCID iDs on behalf of authors.

We look forward to receiving your revised manuscript.

Kind regards,

Shaheer Ansari

Academic Editor

PLOS One

Journal Requirements:

When submitting your revision, we need you to address these additional requirements.

1. Please ensure that your manuscript meets PLOS ONE's style requirements, including those for file naming. The PLOS ONE style templates can be found at

https://journals.plos.org/plosone/s/file?id=wjVg/PLOSOne_formatting_sample_main_body.pdf and

https://journals.plos.org/plosone/s/file?id=ba62/PLOSOne_formatting_sample_title_authors_affiliations.pdf

2. Please note that PLOS One has specific guidelines on code sharing for submissions in which author-generated code underpins the findings in the manuscript. In these cases, we expect all author-generated code to be made available without restrictions upon publication of the work. Please review our guidelines at https://journals.plos.org/plosone/s/materials-and-software-sharing#loc-sharing-code and ensure that your code is shared in a way that follows best practice and facilitates reproducibility and reuse.

3. Thank you for stating the following financial disclosure: [The Major Science and Technology Special Project of Sichuan Province (2024ZDZX0035);].

Please state what role the funders took in the study.  If the funders had no role, please state: ""The funders had no role in study design, data collection and analysis, decision to publish, or preparation of the manuscript.""

If this statement is not correct you must amend it as needed.

Please include this amended Role of Funder statement in your cover letter; we will change the online submission form on your behalf.

4. We note that your Data Availability Statement is currently as follows: [All relevant data are within the manuscript and its Supporting Information files.]

Please confirm at this time whether or not your submission contains all raw data required to replicate the results of your study. Authors must share the “minimal data set” for their submission. PLOS defines the minimal data set to consist of the data required to replicate all study findings reported in the article, as well as related metadata and methods (https://journals.plos.org/plosone/s/data-availability#loc-minimal-data-set-definition).

For example, authors should submit the following data:

- The values behind the means, standard deviations and other measures reported;

- The values used to build graphs;

- The points extracted from images for analysis.

Authors do not need to submit their entire data set if only a portion of the data was used in the reported study.

If your submission does not contain these data, please either upload them as Supporting Information files or deposit them to a stable, public repository and provide us with the relevant URLs, DOIs, or accession numbers. For a list of recommended repositories, please see https://journals.plos.org/plosone/s/recommended-repositories.

If there are ethical or legal restrictions on sharing a de-identified data set, please explain them in detail (e.g., data contain potentially sensitive information, data are owned by a third-party organization, etc.) and who has imposed them (e.g., an ethics committee). Please also provide contact information for a data access committee, ethics committee, or other institutional body to which data requests may be sent. If data are owned by a third party, please indicate how others may request data access.

5. If the reviewer comments include a recommendation to cite specific previously published works, please review and evaluate these publications to determine whether they are relevant and should be cited. There is no requirement to cite these works unless the editor has indicated otherwise.

[Note: HTML markup is below. Please do not edit.]

Reviewers' comments:

Reviewer's Responses to Questions

**Comments to the Author**

1. Is the manuscript technically sound, and do the data support the conclusions?

The manuscript must describe a technically sound piece of scientific research with data that supports the conclusions. Experiments must have been conducted rigorously, with appropriate controls, replication, and sample sizes. The conclusions must be drawn appropriately based on the data presented.

Reviewer #1: Yes

Reviewer #2: Partly

**********

2. Has the statistical analysis been performed appropriately and rigorously?

Reviewer #1: Yes

Reviewer #2: N/A

**********

3. Have the authors made all data underlying the findings in their manuscript fully available?

The PLOS Data policy requires authors to make all data underlying the findings described in their manuscript fully available without restriction, with rare exception (please refer to the Data Availability Statement in the manuscript PDF file). The data should be provided as part of the manuscript or its supporting information, or deposited to a public repository. For example, in addition to summary statistics, the data points behind means, medians and variance measures should be available. If there are restrictions on publicly sharing data—e.g. participant privacy or use of data from a third party—those must be specified.

Reviewer #1: Yes

Reviewer #2: No

**********

4. Is the manuscript presented in an intelligible fashion and written in standard English?

PLOS ONE does not copyedit accepted manuscripts, so the language in submitted articles must be clear, correct, and unambiguous. Any typographical or grammatical errors should be corrected at revision, so please note any specific errors here.

Reviewer #1: Yes

Reviewer #2: No

**********

5. Review Comments to the Author

Please use the space provided to explain your answers to the questions above. You may also include additional comments for the author, including concerns about dual publication, research ethics, or publication ethics. (Please upload your review as an attachment if it exceeds 20,000 characters)

Reviewer #1: This paper presents an improved Back Propagation network model based on multi-feature combinations using Electrochemical Impedance Spectroscopy data to predict the State of Health of lithium-ion batteries. By analyzing the cyclic aging experimental data of four sets of lithium-ion batteries, the Spearman's Rank Correlation Coefficient is applied to identify variables significantly correlated with capacity degradation. The manuscript presents some significant results worthy of publication. It can be revised to be more precise and accurate in its wording. Below are some detailed comments:

1. The novelty of the work should be explained in detail in the Abstract. A more detailed presentation of innovation should be conducted.

2. Please pay more attention to the expression check. Please try to conduct more literature reviews. Please provide a brief presentation of your proposed method.

3. Please highlight the part improved by your research and try to make it more obvious.

4. More recent literature should be cited and analyzed for mathematical analysis, such as Improved hyperparameter Bayesian optimization-bidirectional long short-term memory optimization for high-precision battery state of charge estimation, Improved anti-noise adaptive long short-term memory neural network modeling for the robust remaining useful life prediction of lithium-ion batteries, and Improved multiple feature-electrochemical thermal circuitry modeling of lithium-ion batteries at low temperature with real-time coefficient correction.

5. The writing of the paper should be improved. Please try to improve the content and revise the presentation to clarify the reader's understanding of your idea and make the logic more precise and accurate. Additionally, the innovation and realization should be described in clear terms.

6. The mathematical analysis should be presented more clearly with figures and equations to convey your ideas effectively.

7. Please express your innovation briefly in the conclusion.

8. A comparison with references should be conducted for verification of the advantage.

9. Much of the content is expressed using the traditional methods for this version. Please pay more attention to your content.

Reviewer #2: This paper proposes a battery SOH estimation method based on electrochemical impedance spectroscopy and a backpropagation neural network. However, the manuscript has fundamental shortcomings in terms of novelty, result analysis, and reproducibility.

1. The combination of EIS feature extraction and BP neural networks is already widely used in the literature. This approach is often employed as a baseline method in related studies. The novelty is limited, and the authors have not conducted a comprehensive review of existing EIS based SOH estimation methods.

2. It fails to provide a comprehensive overview of the mainstream directions and methods in current EIS based SOH estimation research.

3. The manuscript confuses physical parameters from equivalent circuit models with geometric features. The proposed features (ohmic resistance, SEI resistance, and charge transfer resistance) are obtained through EIS curve and should be considered geometric features. Although they are related to physical properties, the two concepts should not be used interchangeably.

4. The dataset is extremely small and insufficient. Only two groups of battery data, totaling a few dozen samples, are used as the test set, which cannot support claims of “high accuracy.”

5. The method used to extract geometric features is not clearly described, nor is it specified at which SOC levels the EIS data were collected. The model structure and parameters are also insufficiently detailed, including the number of hidden layers, number of neurons, learning rate, regularization, and number of training epochs.

**********

6. PLOS authors have the option to publish the peer review history of their article (what does this mean?). If published, this will include your full peer review and any attached files.

If you choose “no”, your identity will remain anonymous but your review may still be made public.

**Do you want your identity to be public for this peer review?**  For information about this choice, including consent withdrawal, please see our Privacy Policy.

Reviewer #1: No

Reviewer #2: No

**********

[NOTE: If reviewer comments were submitted as an attachment file, they will be attached to this email and accessible via the submission site. Please log into your account, locate the manuscript record, and check for the action link "View Attachments". If this link does not appear, there are no attachment files.]

To ensure your figures meet our technical requirements, please review our figure guidelines: https://journals.plos.org/plosone/s/figures

You may also use PLOS’s free figure tool, NAAS, to help you prepare publication quality figures: https://journals.plos.org/plosone/s/figures#loc-tools-for-figure-preparation.

NAAS will assess whether your figures meet our technical requirements by comparing each figure against our figure specifications.

# Author comment: Author response to Decision Letter 1

**DOI:** 10.1371/journal.pone.0354706.r002

17 Jun 2026

Response to Reviewer #1

Dear Reviewer,

Thank you very much for your valuable comments and constructive suggestions, which have greatly helped us improve the quality and presentation of this manuscript. We have carefully addressed all your comments point by point, and the revisions are marked in red in the revised manuscript.

Comment 1: The novelty of the work should be explained in detail in the Abstract. A more detailed presentation of innovation should be conducted.

Response:

We thank the reviewer for this suggestion. We have revised the Abstract to explicitly highlight the three main innovations of this work: (1) constructing a multi-feature combination scheme based on both physical and geometric features extracted from EIS data; (2) using Spearman’s Rank Correlation Coefficient (SRCC) to screen features highly correlated with capacity degradation; (3) comparing three intelligent optimization algorithms (GA, PSO, ACO) to optimize the BP network, with ACO and GA achieving the best performance. The revised Abstract is now more concise and clearer about the novelty of the study.

Comment 2: Please pay more attention to the expression check. Please try to conduct more literature reviews. Please provide a brief presentation of your proposed method.

Response:

We have carefully revised the language throughout the manuscript to ensure consistent terminology, clear expressions, and appropriate academic tone. All abbreviations are now defined on first mention. In the Introduction, we have expanded the literature review on EIS-based SOH estimation methods, including single-parameter, ECM-based, and multi-feature/machine learning approaches. We have also added a brief overview of the proposed framework at the end of the Introduction, describing the steps of EIS feature extraction, feature selection, model optimization, and performance evaluation.

Comment 3: Please highlight the part improved by your research and try to make it more obvious.

Response:

To make our contributions clearer, we have added explicit descriptions of our improvements in several key sections:

In the Introduction, we explicitly contrast our multi-feature fusion scheme with traditional single-parameter EIS methods.

In the Feature Extraction section, we have clarified the advantages of combining both physical and geometric features.

In the Model section, we have emphasized how the introduction of intelligent optimization algorithms addresses the local optimum problem of conventional BP networks.

These improvements are marked in red in the revised manuscript.

Comment 4: More recent literature should be cited and analyzed for mathematical analysis, such as Improved hyperparameter Bayesian optimization–bidirectional long short-term memory optimization for high-precision battery state of charge estimation, Improved anti-noise adaptive long short-term memory neural network modeling for the robust remaining useful life prediction of lithium-ion batteries, and Improved multiple feature-electrochemical thermal circuitry modeling of lithium-ion batteries at low temperature with real-time coefficient correction.

Response:

We appreciate this suggestion and have added citations and brief discussions of the recommended papers in the revised manuscript. We have compared our work with these recent studies, noting that while they focus on time-series or thermal modeling, our method leverages EIS multi-features to directly reflect electrochemical aging mechanisms, achieving good interpretability and high accuracy without requiring complex recurrent neural network structures. These discussions have been added in the Introduction.

Comment 5: The writing of the paper should be improved. Please try to improve the content and revise the presentation to clarify the reader's understanding of your idea and make the logic more precise and accurate. Additionally, the innovation and realization should be described in clear terms.

Response:

We have restructured the manuscript to improve logical flow: the Introduction clearly states the research background, problem, and objectives; the Methods section systematically describes feature extraction, feature selection, and model construction; the Results section presents the evaluation of different feature combinations and optimization algorithms; and the Conclusion summarizes the main findings and future work. The innovation and realization of the proposed method are now described in clear, step-by-step terms throughout the paper.

Comment 6: The mathematical analysis should be presented more clearly with figures and equations to convey your ideas effectively.

Response:

We have optimized and adjusted the formulas in the revised manuscript: Formula 1 originally presented in the Introduction has been removed, and we have expanded the literature review on EIS-based research, including relevant work on BO-BiLSTM methods. To improve the transparency and reproducibility of this study, the raw data will be deposited in the PLOS ONE public dataset repository. The BP neural network and optimization algorithms adopted in this work use the default training functions provided by MATLAB. These revisions make the mathematical derivations and algorithmic principles more intuitive, and further enhance the reproducibility of the present study.

Comment 7: Please express your innovation briefly in the conclusion.

Response:

We have revised the Conclusion to begin with a concise statement of the main innovations: (1) EIS multi-feature fusion combining physical and geometric characteristics; (2) SRCC-based feature screening and optimal combination selection; (3) the ACO-BP and the GA-BP network achieving superior prediction performance. The Conclusion now clearly restates the novelty and contributions of the work.

Comment 8: A comparison with references should be conducted for verification of the advantage.

Response:

We have already compared the performance of three optimized models in the results section, and the ACO-BP and the GA-BP network presents competitive advantages. Following the reviewer’s suggestions, we supplemented the detailed parameter configurations of ACO, PSO, GA and the BP network. To ensure fair comparison, we unified the relevant parameter settings of the three optimization algorithms as much as possible before conducting the experiments. We further compared our results with those reported in Reference [11], and the proposed method achieves better performance. Nevertheless, since the complete parameter settings were not fully disclosed in Reference [11], we do not elaborate on this part in the manuscript.

Comment 9: Much of the content is expressed using the traditional methods for this version. Please pay more attention to your content.

Response:

We have significantly reduced the description of traditional BP neural network background and instead expanded the presentation of our own contributions: the EIS multi-feature extraction scheme, SRCC-based feature selection, feature combination comparison, and intelligent optimization of the BP network. The proportion of content dedicated to our innovations has been increased, making the manuscript more focused and highlighting our work more clearly.

Thank you again for your constructive comments, which have greatly improved the quality of this manuscript.

Sincerely,

Fei Chen

Response to Reviewer #2

Dear Reviewer,

Thank you very much for your careful review and valuable suggestions, which have helped us greatly improve the novelty, clarity, and reproducibility of this manuscript. We have carefully addressed all your comments point by point, and the revisions are marked in red in the revised version.

Comment 1: The combination of EIS feature extraction and BP neural networks is already widely used in the literature. This approach is often employed as a baseline method in related studies. The novelty is limited, and the authors have not conducted a comprehensive review of existing EIS-based SOH estimation methods.

Response:

We sincerely appreciate the reviewer’s professional and insightful comments on the novelty and literature review. We fully acknowledge that the combination of EIS feature extraction and BP neural network has been applied as a common baseline in battery SOH estimation. However, based on the comprehensive review of the latest representative studies (Refs. [11–16]), our work presents clear and substantial improvements in feature engineering, feature selection strategy, and model optimization mechanism, which effectively enhance the prediction accuracy, interpretability, and stability compared with traditional EIS–BP frameworks.

1. Comprehensive review of existing EIS‑based SOH estimation methods

As summarized in the revised Introduction, mainstream EIS‑driven SOH approaches can be categorized into three branches:

Equivalent circuit model (ECM) parameter‑based methods

Ref. [11] systematically analyzed various ECMs for lithium‑ion batteries using EIS, focusing on circuit topology and parameter identification, but only used single physical parameters (e.g., ohmic resistance, charge‑transfer resistance) for state evaluation, lacking multi‑feature fusion.

Ref. [12] achieved rapid SOH estimation via automated EIS and ECMs, but still relied on conventional circuit fitting parameters without geometric feature mining.

Relaxation time distribution (DRT)‑based methods

Ref. [13] integrated DRT parameters with LSTM network for SOH prediction, which improves time‑series feature learning, but depends on complex DRT calculation and cannot explicitly distinguish physical and geometric characteristics of EIS.

Direct EIS feature + machine learning methods

Refs. [14, 15] adopted machine learning models combined with EIS features for SOH estimation, while mostly using shallow networks or fixed feature sets without targeted optimization for BP local optimum issue.

Ref. [16] proposed Bayesian optimization‑BiLSTM for SOC estimation, which belongs to time‑series sequential modeling, significantly different from our static EIS feature‑based SOH prediction framework.

2. Novelty and improvements of this work

Compared with the above studies, our method breaks through the limitations of traditional EIS–BP framework in the following aspects:

Dual‑type multi‑feature fusion

Instead of single ECM parameters or simple impedance values, we extract physical parameters (from ECM fitting) and geometric features (from Nyquist plot) simultaneously, forming a more comprehensive feature set that reflects both electrochemical mechanism and curve morphology.

SRCC‑driven feature screening

We use Spearman’s rank correlation coefficient to quantitatively measure feature‑degradation correlation and construct optimal feature combinations, avoiding redundant or weakly correlated features that are common in conventional methods.

optimized BP network

After comparing multiple schemes, this study adopts Ant Colony Optimization (ACO) and Genetic Algorithm (GA) to optimize the learning rate and regularization coefficient of the BP neural network. This approach addresses the problem that conventional BP neural networks tend to fall into local optima, and the optimized model achieves higher accuracy than the original BP network and the PSO-optimized BP network (PSO-BP).

Comment 2: It fails to provide a comprehensive overview of the mainstream directions and methods in current EIS-based SOH estimation research.

Response:

We appreciate this suggestion. We have expanded the Introduction to provide a systematic overview of the mainstream directions in EIS-based SOH estimation:

(1) Single-parameter methods using only ohmic resistance or charge transfer resistance;

(2) Equivalent circuit model (ECM)-based methods that fit impedance spectra with circuit elements;

(3) Multi-feature and machine learning-based methods that combine EIS features with data-driven models.

We have also clarified the position of our work in this context, showing that our multi-feature fusion and optimized BP network belong to the third category but with specific improvements in feature design and model optimization.

Comment 3: The manuscript confuses physical parameters from equivalent circuit models with geometric features. The proposed features (ohmic resistance, SEI resistance, and charge transfer resistance) are obtained through EIS curve and should be considered geometric features. Although they are related to physical properties, the two concepts should not be used interchangeably.

Response:

We thank the reviewer for pointing out this confusion. We have revised the manuscript to clearly distinguish and define the two types of features:

Physical parameters: Obtained by fitting the EIS curve with an equivalent circuit model, including ohmic resistance RS, SEI film resistance RSEI, and charge transfer resistance RCT . These parameters have clear physical meanings related to internal electrochemical processes.

Geometric features: Directly extracted from the Nyquist plot, such as the center coordinates and radius of the two arcs, the horizontal difference between arc centers, and the maximum imaginary part value. These features describe the shape of the impedance curve.

We explicitly state that the multi-feature combination in this work integrates both types of features to comprehensively characterize battery aging, and the two terms are no longer used interchangeably.

Comment 4: The dataset is extremely small and insufficient. Only two groups of battery data, totaling a few dozen samples, are used as the test set, which cannot support claims of “high accuracy.”

Response:

Thank you for this valuable comment. We have supplemented and revised the description of the dataset in the revised manuscript.

In this study, experimental data collected from the same type of lithium-ion batteries under four different operating conditions are adopted. The total number of samples is well over 200, covering the full aging cycle of the batteries from the fresh state to an SOH of 80%. The sufficient sample size reduces errors caused by random data splitting and improves the reliability of experimental results.

In addition, we have revised the overstated expressions in the original text. The relevant statement is updated to: Under the experimental conditions of this work, the proposed method achieves favorable estimation accuracy for battery aging data. We also acknowledge that further validation using datasets from various types of batteries is required in future research, which has been included in our research plan.

Comment 5: The method used to extract geometric features is not clearly described, nor is it specified at which SOC levels the EIS data were collected. The model structure and parameters are also insufficiently detailed, including the number of hidden layers, number of neurons, learning rate, regularization, and number of training epochs.

Response:

We thank the reviewer for pointing out the missing methodological details, and we have made corresponding supplements and revisions in the revised manuscript:

(1) The structure and parameter settings of the BP neural network have been fully supplemented.

(2) We have added the parameter configurations of three optimization algorithms, including Genetic Algorithm (GA), Particle Swarm Optimization (PSO) and Ant Colony Optimization (ACO).

(3) The data source and experimental test methods refer to Reference [24]: Zhang Y W, Tang Q C, Zhang Y, et al. Identifying degradation patterns of lithium ion batteries from impedance spectroscopy using machine learning. Nature Communications, 11(1): 1706.

(4) The extraction of EIS features follows the method presented in Reference [11]: Westerhoff U, Kurbatov D, Kroger T, Sauer D U. Analysis of lithium-ion battery models based on electrochemical impedance spectroscopy. Energy Technology, 2016, 4(10): 1228-1238. https://doi.org/10.1002/ente.201600154.These additions improve the

reproducibility of our work, and all details are clearly marked in red.

Thank you again for your valuable comments and suggestions, which have significantly improved the quality and clarity of this manuscript.

Sincerely,

Fei Chen

Attachment Submitted filename: Response to the Reviewer 2.docx

# Peer review: Decision Letter 1

**DOI:** 10.1371/journal.pone.0354706.r003

**Authors:** Shaheer Ansari

13 Jul 2026

Research on Predicting the State of Health of Lithium-Ion Batteries via Back Propagation Network Based on Multi-Feature Combination of Electrochemical Impedance Spectroscopy

PONE-D-26-11604R1

Dear Dr. fei,

We’re pleased to inform you that your manuscript has been judged scientifically suitable for publication and will be formally accepted for publication once it meets all outstanding technical requirements.

Within one week, you’ll receive an e-mail detailing the required amendments. When these have been addressed, you’ll receive a formal acceptance letter and your manuscript will be scheduled for publication.

An invoice will be generated when your article is formally accepted. Please note, if your institution has a publishing partnership with PLOS and your article meets the relevant criteria, all or part of your publication costs will be covered. Please make sure your user information is up-to-date by logging into Editorial Manager at Editorial Manager® and clicking the ‘Update My Information' link at the top of the page. For questions related to billing, please contact billing support.

If your institution or institutions have a press office, please notify them about your upcoming paper to help maximize its impact. If they’ll be preparing press materials, please inform our press team as soon as possible -- no later than 48 hours after receiving the formal acceptance. Your manuscript will remain under strict press embargo until 2 pm Eastern Time on the date of publication. For more information, please contact onepress@plos.org.

Kind regards,

Shaheer Ansari

Academic Editor

PLOS One

**********

# Editor report: Acceptance letter

**DOI:** 10.1371/journal.pone.0354706.r004

**Authors:** Shaheer Ansari

PONE-D-26-11604R1

PLOS One

Dear Dr. Chen,

I'm pleased to inform you that your manuscript has been deemed suitable for publication in PLOS One. Congratulations! Your manuscript is now being handed over to our production team.

At this stage, our production department will prepare your paper for publication. This includes ensuring the following:

* All references, tables, and figures are properly cited

* All relevant supporting information is included in the manuscript submission,

* There are no issues that prevent the paper from being properly typeset

You will receive further instructions from the production team, including instructions on how to review your proof when it is ready. Please keep in mind that we are working through a large volume of accepted articles, so please give us a few days to review your paper and let you know the next and final steps.

Lastly, if your institution or institutions have a press office, please let them know about your upcoming paper now to help maximize its impact. If they'll be preparing press materials, please inform our press team within the next 48 hours. Your manuscript will remain under strict press embargo until 2 pm Eastern Time on the date of publication. For more information, please contact onepress@plos.org.

You will receive an invoice from PLOS for your publication fee after your manuscript has reached the completed accept phase. If you receive an email requesting payment before acceptance or for any other service, this may be a phishing scheme. Learn how to identify phishing emails and protect your accounts at https://explore.plos.org/phishing.

If we can help with anything else, please email us at customercare@plos.org.

Thank you for submitting your work to PLOS ONE and supporting open access.

Kind regards,

PLOS ONE Editorial Office Staff

on behalf of

Dr. Shaheer Ansari

Academic Editor

PLOS One
